# Case Report: Optical genome mapping enables identification of complex balanced chromosomal rearrangements

**DOI:** 10.3389/fgene.2025.1555485

**Published:** 2025-05-22

**Authors:** Xiaohang Hu, Jing Guo, Haiyang Sang, Jinyan Yan, Hong Chang, Ting Liu, Haixin Dong, Min Kong, Yanjun Tian, Liqing Jiang

**Affiliations:** ^1^ Department of Medical Laboratory, Affiliated Hospital of Jining Medical University, Shandong Key Laboratory of Multi-disciplinary Molecular Diagnosis Precision Medicine, Jining, Shandong, China; ^2^ Department of Medical Laboratory, Disease Prevention and Control Center of Jining Rencheng District, Jining, Shandong, China; ^3^ Medical Laboratory of Jining Medical University, Lin He’s Academician Workstation of New Medicine and Clinical Translation in Jining Medical University, Jining Medical University, Jining, Shandong, China

**Keywords:** optical genome mapping, high-resolution karyotyping analysis, chromosomal microarray analysis, balanced reciprocal rearrangements, complex chromosomal rearrangements

## Abstract

**Introduction:**

Individuals with balanced chromosomal rearrangements are at an increased risk for infertility, recurrent miscarriages, and the birth of infants with congenital malformations. Traditional cytogenetic techniques are limited by their low resolution, whereas optical genome mapping offers enhanced capabilities for detecting chromosomal rearrangements and determining genomic localization and orientation. This study sought to evaluate the efficacy of optical genome mapping in identifying complex balanced chromosomal rearrangements that may contribute to fertility challenges.

**Case presentation:**

A 21-year-old Asian female patient with a history of recurrent abortions was included in the study. Peripheral blood samples were collected for high-resolution karyotyping, chromosomal microarray analysis, and optical genome mapping. The high-resolution karyotype analysis identified complex chromosomal abnormalities. Optical genome mapping has revealed additional cryptic chromosomal aberrations, such as ins (2; 12) (p16.1; q12q12), inv (6) (q21q21), and inv (12) (q12q12), offering a novel perspective on this case. Notably, the disrupted genes, including *CRIM1*, *MUC19*, and *PRDM1*, have not been classified as pathogenic by existing databases.

**Conclusion:**

This study underscores the capability of optical genome mapping to deliver comprehensive and precise information. It is anticipated that optical genome mapping will emerge as a valuable cytogenetic tool within clinical genetic methodologies, providing new references and insights for clinical practice in the future.

## Introduction

Complex chromosomal rearrangements (CCRs) play a critical role in various human genetic disorders by disrupting protein-coding genes and cis-regulatory elements. These rearrangements encompass a range of distinct structural variations on the same allele within a single mutational signature event (Collins et al., 2020). Balanced reciprocal rearrangements (BCRs) occur due to the random *de novo* breakage and subsequent rejoining of two or more chromosomes. The incidence rate of BCRs is approximately 7% among infertile couples, a figure that is significantly elevated compared to the general human population (0.14%) (Kasikova et al., 2012). Most carriers of BCRs exhibit normal phenotypes, as the majority of breakpoints (BPs) occur in intergenic or noncoding regions, where the disruption has minimal interference with gene expression (Carvalho and Lupski, 2016). However, these individuals face an increased risk of recurrent miscarriages and the birth of newborns with chromosomal imbalances (Chow et al., 2020). Research indicates that the likelihood of identifying normal or balanced blastocysts in patients with CCRs is less than 6%, while the probability of achieving pregnancy is below 4%. These factors may contribute to a diminished number of transplantable embryos (L. Hu et al., 2018). This may manifest as implantation failure, miscarriage of established pregnancies, or the birth of infants with chromosomal syndromes, which poses significant concerns for affected couples (Wilch and Morton, 2018). In a study involving 300 couples experiencing recurrent miscarriages, chromosomal abnormalities were identified in 26 cases, representing 8.7% of the sample. Among these, structural anomalies being the most prevalent at 57.7%, followed by numerical chromosomal aberrations at 42.3% (Yildirim et al., 2019). Genetic analyses of BCRs associated with a normal phenotype hold significant clinical implications for fertility counseling and the prevention of genetic disorders in offspring.

Routine cytogenetic analyses are essential for identifying genetic biomarkers relevant to clinical diagnosis, however, each testing method presents specific limitations. Karyotyping, recognized as a fundamental first-line approach, is capable of identifying both numerical and structural chromosomal aberrations. Nonetheless, it is characterized by certain drawbacks, including a time-intensive process, limited microscopic resolution (averaging between 5 and 10 Mb), and a relatively low overall diagnostic yield (Hofherr et al., 2011; Dremsek et al., 2021; MacLeod and Drexler, 2013). Copy number variation sequencing (CNV-seq) and chromosomal microarray analysis (CMA) are capable of identifying variations as small as a few kilobases (Kb). However, these methods have limitations in detecting lower-proportion mosaicism and BCRs (Miller et al., 2010; Waggoner et al., 2018). Fluorescence *in situ* hybridization (FISH) serves as a viable method for confirming suspected cryptic BCRs. However, its application is constrained by challenges in determining the precise localization beforehand and the complexities involved in assigning specific fluorescent probes. Consequently, FISH is not appropriate for comprehensive gene detection (Cui et al., 2016). Whole genome sequencing (WGS) facilitates the identification of structural rearrangements and nucleotide variations within a single experimental framework, generating significant interest in its potential as a primary diagnostic tool (Lionel et al., 2017). However, its implementation is hindered by challenges such as limited read length, high costs, and reduced sensitivity in repetitive genomic regions (Savara et al., 2021). Phasing multiple rearrangement breakpoints in complex B Cell receptor structures using WGS can be a formidable task (Dong et al., 2017). Consequently, there is a pressing need for a rapid and reliable method to identify BCRs during clinical testing.

Optical genome mapping (OGM) represents a sophisticated, high-throughput cytogenomic methodology that utilizes imaging techniques to analyze exceptionally long linear single DNA molecules. OGM offers enhanced sensitivity and specificity in the detection of various structural variants (SVs), encompassing aneuploidies, deletions, duplications, inversions, insertions, translocations, and the absence of heterozygosity (AOH) (Mantere et al., 2021). The capabilities of preamplification-free techniques and high-resolution analysis are enhanced through the fluorescent labeling of specific sequence motifs (Sanders et al., 2019; Chan Saki et al., 2018). OGM offers significant advantages, including improved turnaround times and cost-effective generation of 300–500 × genome-wide coverage. Recent studies have demonstrated the efficacy of OGM in identifying a broad spectrum of genetic disorders associated with cancers (Neveling et al., 2021; Zhang et al., 2023a; Sahajpal et al., 2021). The finding indicates that OGM demonstrated complete concordance with cytogenetic assays in detecting genomic chromosomal aberrations across 85 individuals (Mantere et al., 2021). Furthermore, OGM has been effectively employed to elucidate the relationship between genes and phenotypes based on breakpoint locations. Additionally, OGM possesses the capability to identify intricate genetic abnormalities and facilitates the characterization of breakpoints (Wang et al., 2020; Chan EvaKF. et al., 2018; Jain et al., 2016).

Significant challenges have thus far constrained the objective assessment of BCRs. We uncovered a complex BCRs by OGM analysis, which was discribed as 46,XX,ins (6; 2) (q21; p16.1p22.2) ins (2; 12) (p16.1; q12q12) inv (6) (q21q21) t (6; 12) (q21; q12) inv (12) (q12q12), which has not been previously reported. This study aims to investigate the potential of OGM in detecting complex BCRs in the absence of various clinical indications, offering valuable references and insights for future clinical diagnoses.

## Case description

A 21-year-old Asian woman participated in this study. She was diagnosed with pregnancy at the Affiliated Hospital of Jining Medical University. Eclampsia assessment revealed a high risk, with a Placental Growth Factor Multiple Of Median of 0.27. Additionally, mid-trimester Down syndrome screening indicated a heightened risk of open neural tube malformation, with a Multiple Of Median of 2.407. At 19 weeks of gestation, the ultrasound findings indicated the presence of multiple fetal developmental anomalies, including a reduced size of the cerebellar vermis and a cystic echo observed in the cerebellar bulbous cisterna. The results of the auxiliary examinations were normal.

**FIGURE 1:**
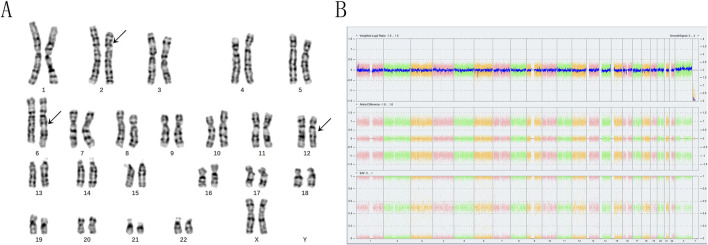
The karyotyping and chromosomal microarray analyses of the subject revealed significant findings. **(A)** The arrows indicate the presence of abnormal chromosomes. The karyotyping analysis demonstrated the insertion of the p16p22 segment from chromosome 2 into the q21 region of chromosome 6, as well as a translocation between the q21 segment of chromosome 6 and the q13 segment of chromosome 12. This was characterized as 46,XX,ins (6; 2) (q21; p16p22) t (6; 12) (q21; q13). **(B)** In contrast, the chromosomal microarray analysis yielded normal results.

The family opted for labor induction following an abnormal fetal ultrasound examination. CNV-seq (Copy Number Variation Sequencing) genetic analysis of the aborted tissue revealed a 22.5 Mb duplication in the p22.3p16.1 segment of chromosome 2 [seg (GRch38) dup (2) (p22.3p16.1) Chr2:g36,500,001-59,000,001dup], which is classified as pathogenic According to the ACMG guidelines (Riggs, E. R., et al. (2019). Genet Med. doi:10.1038/s 41436-019-0686-8. Relevant databases such as ClinGen, DECIPHER, OMIM, ClinVar and PubMed and others if required suggest that the identified duplicated fragment is linked to various clinical manifestations, including intellectual disability, growth retardation, neonatal hypotonia, syndactyly, gastroesophageal reflux, hydrocephalus, anal atresia, and short stature. Given the significant size of the fragment, the couple was advised to collect anticoagulant peripheral blood to undergo high-resolution karyotyping analysis and CMA. The subject exhibited normal phenotypes, and her CMA results indicated no imbalanced structural variations according to the American College of Medical Genetics and Genomics (ACMG) guidelines. Consequently, we hypothesized that she was a carrier of BCRs. To validate this hypothesis, we employed high-resolution karyotyping according to the International System for Human Cytogenetic Nomenclature (ISCN) 2020 and OGM based on the GRCh38 human reference genome. The subject’s husband’s karyotype was characterized as 46,XY (Figure 1A), with normal CMA results (Figure 1B).

## Results

**FIGURE 2:**
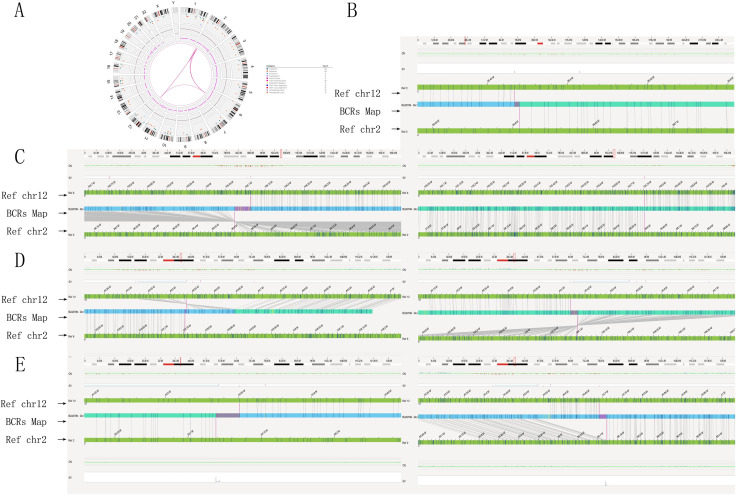
Genome-wide visualization of optical genome mapping reveals significant chromosomal structural rearrangements. **(A)** The Circos plot integrates all identified chromosomal alterations, with the default view organized from the outer to the inner circle: cytoband locations numbered 1 to 24 (1–22, XX). Color-coded interstitial structural variations are highlighted at specific locations, alongside observed copy number changes for each chromosome or region, illustrating translocations, insertions, and inversions represented as lines. Notably, the Circos plot indicates connections between chromosomes 2, 6, and 12 at genomic loci, depicted with red lines. **(B)** The genome map view illustrates the inversion of chromosome 2p16.1p22.2. **(C)** The genome map view details the breakpoint junction of an insertion, ins (6; 2) (q21; p16.1p22.2). **(D)** The genome map view presents the breakpoint junction of a translocation, t (6; 12) (q21; q12). **(E)** The genome map view highlights the breakpoint junction of another insertion, ins (2; 12) (p16.1; q12q12). Abbreviations used include INS for insertion, DEL for deletion, INV for inversion, DUP for duplication, and TRA for translocation.

The karyotyping results confirmed that the subject was indeed a carrier of BCRs, characterized as 46,XX, with a specific rearrangement involving chromosomes 2, 6, and 12. Specifically, the p16p22 segment of chromosome two was inserted into the q21 segment of chromosome 6, accompanied by a mutual translocation between the q21 segment of chromosome six and the q13 segment of chromosome 12. This rearrangement was described as 46,XX,ins (6; 2) (q21; p16p22) t (6; 12) (q21; q13) (Figure 2A).

OGM identified a reciprocal translocation between chromosomes 6q21 and 12q12, as well as the insertion of a 2p segment into 6q21, which closely aligned with the findings from karyotyping analysis (Figures 2C,D). Furthermore, the insertion segment of chromosome two was refined from p16p22 to p16.1p22.2 (Figure 2B). OGM also provided detailed information regarding additional fragments, including fus (2; 2) (p22.2; p16.1) involving a 22.1 Mb segment of chromosome 2 (BP: 36,466,829-58,603,981) (Figure 2B), ins (2; 12) (p16.1; q12q12) with a 141 Kb segment of chromosome 12 (BP: 40,550,771-40,691,368) and chromosome 2 (BP: 56,119,045-56,121,630) (Figure 2E), inv (6) (q21q21) with a 359 Kb segment of chromosome 6 (BP: 105,753,796-106,102,619) (Figure 2D), and inv (12) (q12q12) with a 115 Kb segment of chromosome 12 (BP: 40,550,771-40,691,368) (Figure 2D). Additionally, the orientations of the rearranged chromosomes were determined: the p22.2p16.1 segment of chromosome two was reversed and inserted into the q21 segment of chromosome 6, while the q12q12 segment of chromosome 12 was positively inserted into the p16.1 segment of chromosome 2. The refinement of breakpoints and the orientation of segments significantly contributed to the elucidation of the chromosome rearrangement pattern. The findings from the OGM analysis were characterized by the chromosomal alterations 46,XX,ins (6; 2) (q21; p16.1p22.2) ins (2; 12) (p16.1; q12q12)inv (6) (q21q21) t (6; 12) (q21; q12)inv (12) (q12q12). Detailed breakpoint information is shown in Table 1.

**TABLE 1:** Detailed characterisations of the rearrangements on chromosomes 2, 6, and 12 by Bionano Access Software.

Type	Chr Involved#1	Chr Involved#2	Breakpoint#1 (bp)	Breakpoint#2 (bp)	ISCN
Transl	2	2	36,466,829	58,603,981	ogm [GRCh38]fus (2; 2) (p22.2; p16.1)
Transl	2	6	36,507,200	106,113,348	ogm [GRCh38]t (2; 6) (p22.2; q21)
Transl	2	6	58,598,126	105,752,975	ogm [GRCh38]t (2; 6) (p16.1; q21)
Transl	2	12	56,119,045	40,550,771	ogm [GRCh38]t (2; 12) (p16.1; q12)
Transl	2	12	56,121,630	40,691,368	ogm [GRCh38]t (2; 12) (p16.1; q12)
Transl	6	12	105,753,796	40,536,406	ogm [GRCh38]t (6; 12) (q21; q12)
Transl	6	12	106,102,619	40,421,695	ogm [GRCh38]t (6; 12) (q21; q12)

Chr, chromosome; bp, base pair; Transl, translocation.

## Discussion

CCRs are known to encompass both balanced and imbalanced categories, which can lead to a range of phenotypic outcomes. Additionally, BCRs may result in congenital anomalies in the absence of identifiable causative agents, indicating potential disruptions in gene function and long-range regulatory interactions (Sanders et al., 2019; Sinnerbrink et al., 2013). While the majority of BCRs carriers do not exhibit clear clinical phenotypes, there is a significant increase in the risk of spontaneous abortion, infertility, or the birth of abnormal offspring (Liao et al., 2014), the refinement of cryptic BCRs presents a significant challenge.

In this study, high-resolution karyotyping indicated two breakpoints (2p16, 2p22) on chromosome 2, one breakpoint (6q21) on chromosome 6, and one breakpoint (12q13) on chromosome 12. Balanced rearrangements may not necessarily correlate with abnormal phenotypes; however, certain reverse transfers can alter the orientation of repetitive DNA, leading to the formation of inverted loops during meiosis, which results in a 50% probability of producing imbalanced gametes, characterized by partial duplications and deletions. Consequently, there is an elevated risk of genetic diseases in the offspring of carriers with inversions (Watson et al., 2014; Antonacci et al., 2010).

The precise region and orientation of rearrangements in BPs are crucial for elucidating the genetic underpinnings of diseases. However, existing molecular technologies are inadequate for accurately determining the location and orientation of abnormal segments that disrupt or fuse genes. OGM demonstrates the ability to detect significant types of chromosomal aberrations, facilitating the identification of genes located within breakpoint intervals, which can enhance the clinical interpretation of genetic disorders, particularly concerning intragenic rearrangements in prenatal diagnosis. Furthermore, OGM achieves 100% concordance with karyotype analysis for all aberrations involving non-centromeric breakpoints (Sahajpal et al., 2021; Mantere et al., 2021; Wang et al., 2020).

**FIGURE 3:**
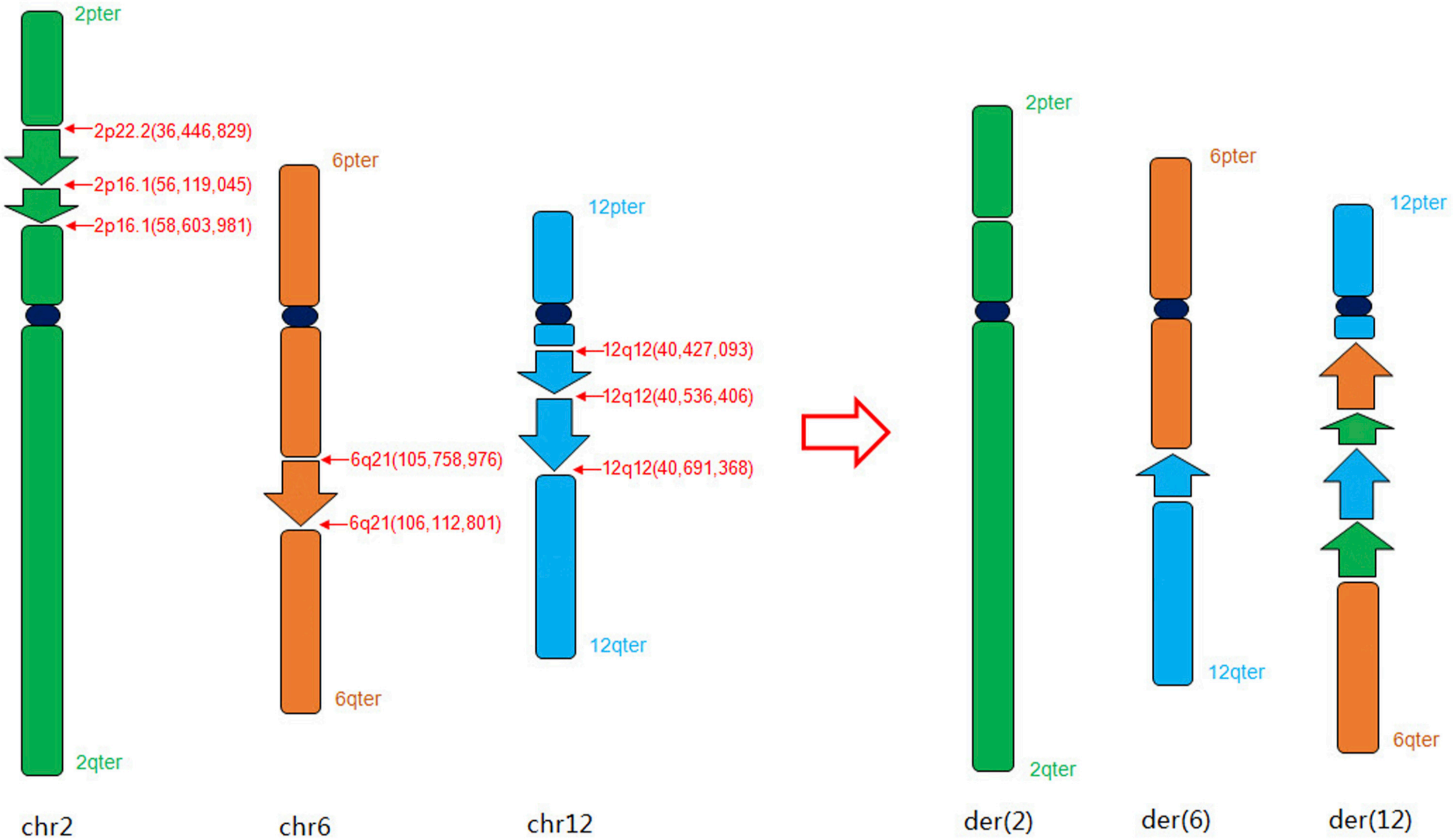
Schematic diagram of the complex chromosome rearrangement pattern in chromosomes 2, 6 and 12 based on OGM analysis.

In our study, The findings of OGM, which were challenging to distinguish through karyotyping, provide new insights into this case. Additionally, the orientations of the rearranged chromosomes were determined: the p22.2p16.1 segment of chromosome two was reversed and inserted into the q21 segment of chromosome 6, while the q12q12 segment of chromosome 12 was positively inserted into the p16.1 segment of chromosome 2. The refinement of breakpoints and the orientation of segments significantly contributed to the elucidation of the chromosome rearrangement pattern. Collectively, our results suggest that OGM demonstrates superior potential and enhanced resolution in identifying specific breakpoints (Table 1) and the orientations of abnormal chromosomal segments, aligning with the observations made by Mantere T. et al. (Mantere et al., 2021). According to the GRCh38 assembly, the *CRIM1* gene was disrupted in the region of chromosome 2 (BP: 36,466,829-36,507,200), the *MUC19* gene was disrupted in the region of chromosome 12 (BP: 40,550,771-40,421,695), and the *PRDM1* gene was disrupted in the region of chromosome 6 (BP: 105,752,975-106,102,619). However, no variants with established pathogenic significance for the genes have been reported. Based on the OGM analysis, we have simulated a schematic diagram of the rearrangement occurring in this case (Figure 3). OGM represents a cutting-edge methodology focused on the manipulation of megabase-length DNA molecules to develop a “fluorescent barcode DNA map.” This innovative approach integrates microfluidics, high-resolution microscopy, and automated image analysis, facilitating high-throughput whole-genome imaging and *de novo* assembly (Sahajpal et al., 2021). The data analysis of OGM employs two advanced pipelines that demonstrate heightened sensitivity for detecting large segments based on coverage depth, as well as small CNVs through the comparative analysis of assembly maps against reference maps. OGM offers significant advantages in identifying previously unrecognized clinically relevant SVs and accurately determining the location and orientation of BPs (P. Hu et al., 2024; Zhang et al., 2023b). The study conducted by Mantere et al. (2021) demonstrated that OGM achieved a remarkable 100% consistency with standard tests in identifying known chromosomal aberrations across 85 blood or cultured cell samples. The study demonstrated a 100% concordance between OGM and karyotype analysis and/or CMA, indicating the efficacy of OGM in identifying genomic alterations in prenatal samples (Goumy, 2023).

OGM was originally primarily used in the diagnosis of hematological diseases (Levy et al., 2022; Nilius-Eliliwi et al., 2023) as well as germline SVs of individual study participants (Barseghyan et al., 2018; Du et al., 2018) and rare diseases (Shieh et al., 2021). Subsequently it was applied to the prenatal and *postpartum* diagnosis (Sahajpal et al., 2021; P; Hu et al., 2024; Zhang et al., 2023a; Goumy, 2023). Researches (Zhang et al., 2023a; Mantere et al., 2021) indicate that OGM is recognized as a precise and thorough technique for identifying cryptic balanced translocations. This capability is instrumental in the genetic diagnosis of patients experiencing unexplained recurrent abortion or infertility. Furthermore, OGM has the potential to forecast disruptions in genes caused by breakpoints, which may lead to the formation of fusion genes (Halgren et al., 2018). The destruction of known pathogenic genes may result in structural abnormalities that can subsequently give rise to clinical syndromes, attributable to inadequate haploid function of the associated genes (Lee et al., 2020; Schluth-Bolard et al., 2019). The study (Wang et al., 2020) identified breakpoint regions in nine subfertile patients with various balanced reciprocal translocations using OGM. It was determined that four disrupted genes, specifically *NUP155*, *FNVDC3A*, *DPY19L1*, and *BAI3*, are associated with male infertility. Additionally, three genes are currently no definitive pathogenic reports, which may account for the normal phenotype observed in the individual. To date, there have been no documented cases involving ins (6; 2), inv (6) (q21q21), or inv (12) (q12q12). Over the past 3 decades, only ten cases of t (6; 12) have been reported, with the involved sites differing and potentially associated with other chromosomal abnormalities (Vandeweyer et al., 2012; Bernheim et al., 2007; Bianco et al., 2011; Coccé et al., 2016), all of which exhibited a variety of clinical manifestations, while other instances may remain unreported due to the absence of identifiable clinical phenotypic abnormalities.

However, OGM presents certain limitations. Primarily, the absence of a comprehensive human reference genome, coupled with the presence of excessively long repeat sequences in the centromeric regions, hinders OGM’s ability to accurately detect balanced structural variations, breakpoints, and fusion sites within structural heterochromatin areas, including centromeres and the short arms of telomeric chromosomes (Qu et al., 2023). The successful completion of telomere-to-telomere genome assembly and the subsequent gap filling of the human reference genome offer promising prospects for overcoming the limitations associated with OGM (Gershman et al., 2022). A recent study indicates that the integration of sequencing techniques and OGM could facilitate the comprehensive assembly of the human X chromosome from telomere to telomere (Miga et al., 2020). Secondly, it is essential to provide specific technical support to ensure the accuracy of the BPs identified by OGM at the single nucleotide level. Furthermore, the relationship between the newly identified variations and the corresponding phenotypes requires additional verification.

## Conclusion

The sensitivity of OGM in this study surpasses that of other established molecular technologies for detecting large-fragment translocations and small-fragment CNVs inversions. Furthermore, OGM offers specific breakpoint information for complex structural aberrations and can predict the potential destruction of breakpoint genes, thereby serving as a valuable reference for disease diagnosis, prevention, and assisted reproduction in patients with structural variations. Nonetheless, the high costs and the requirement for more sophisticated testing techniques and analytical processes currently limit its widespread application in clinical practice. Looking ahead, OGM is anticipated to become a widely adopted tool in clinical settings.

## Data Availability

The original contributions presented in the study are included in the article, further inquiries can be directed to the corresponding authors.
